# Supplementary material for: Supramolecular metallic foams with ultrahigh specific strength and sustainable recyclability
Source: Nat Commun. 2024 May 29;15:4553. doi: 10.1038/s41467-024-49091-6 (PMC11137098; doi:10.1038/s41467-024-49091-6)
Supplement: Supplementary file 1 — Supplementary Information [file 41467_2024_49091_MOESM1_ESM.pdf]

Supplementary Information

Supramolecular Metallic Foams with Ultrahigh Specific Strength and Sustainable Recyclability

*Xin Yang, Xin Huang, Xiaoyan Qiu, Quanquan Guo and Xinxing Zhang**

X. Yang, X. Huang, X. Qiu and Prof. X. Zhang

State Key Laboratory of Polymer Materials Engineering, Polymer Research Institute, Sichuan University, Chengdu 610065, China

E-mail: xxzwwh@scu.edu.cn

Q. Guo

Max Planck Institute of Microstructure Physics, Halle (Saale) 06120, Germany

Supplementary Figures

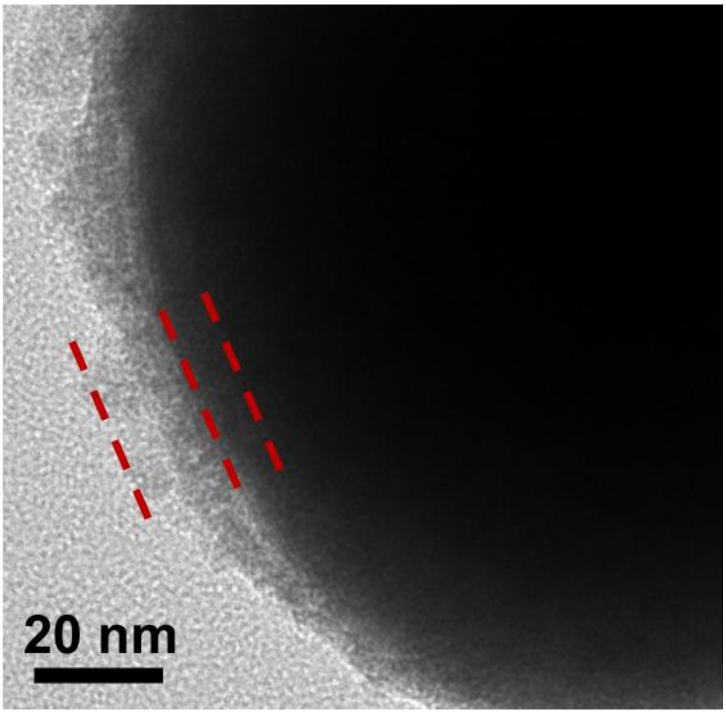

Supplementary Figure 1. High-resolution TEM image of the core–shell structure

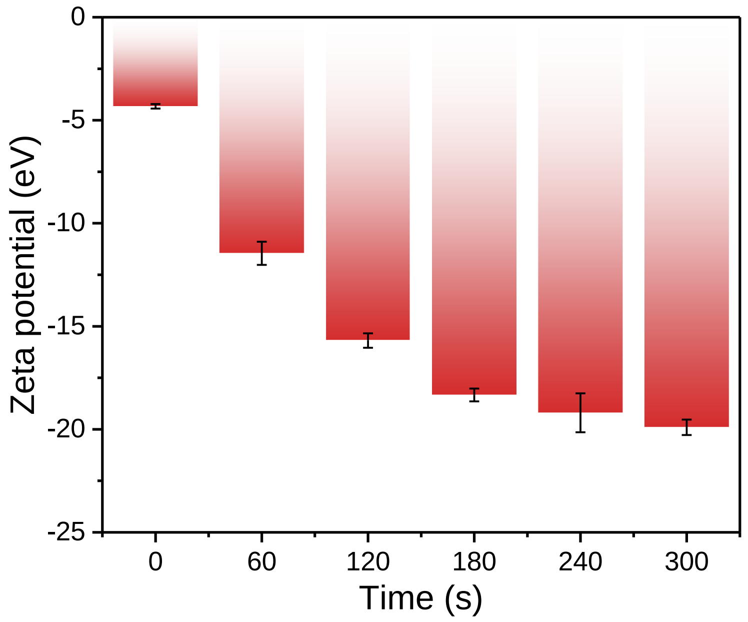

Supplementary Figure 2. Zeta potential of core-shell nanostructure under different time of co-sonication; three measurements were conducted for each data point with the error bars corresponding to the standard deviation.

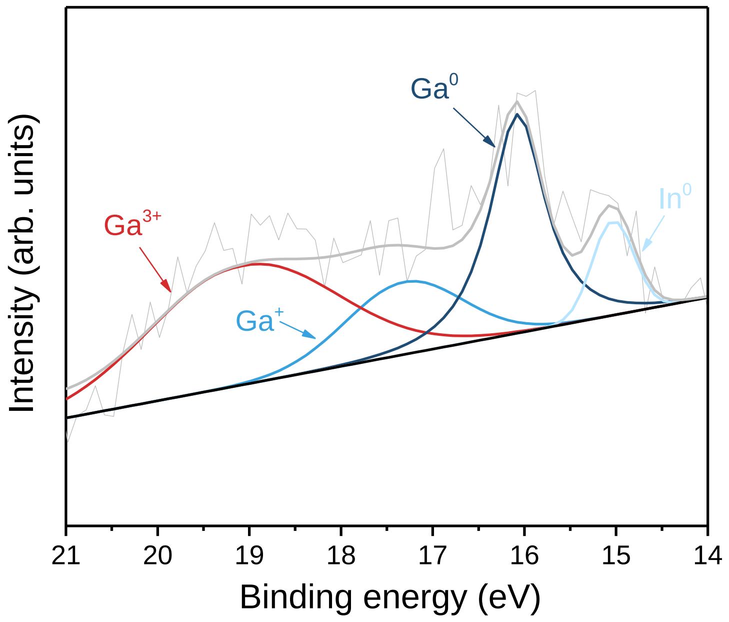

Supplementary Figure 3. Ga 3*d* XPS spectrum of SMF.

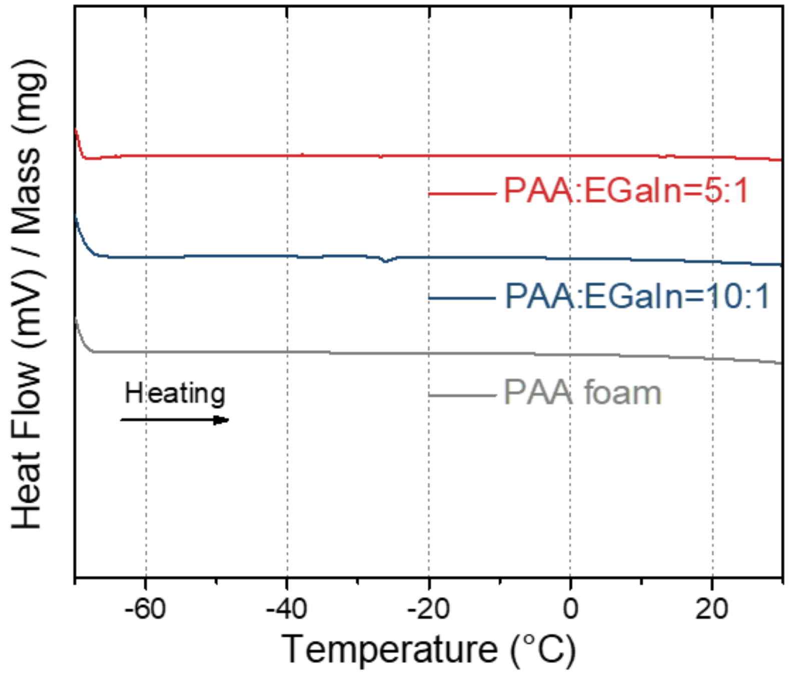

Supplementary Figure 4. DSC curves of SMFs (10 wt% and 20 wt% of EGaIn) and PAA foam during heating process from -70 to 40 ˚C.

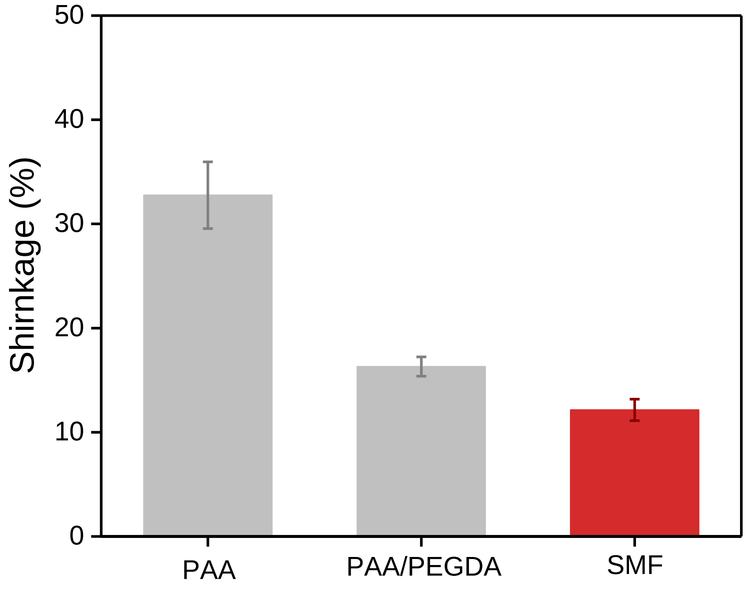

Supplementary Figure 5. Shrinkage of PAA, PAA/PEGDA and SMF after freeze-drying; three measurements were conducted for each data point with the error bars corresponding to the standard deviation.

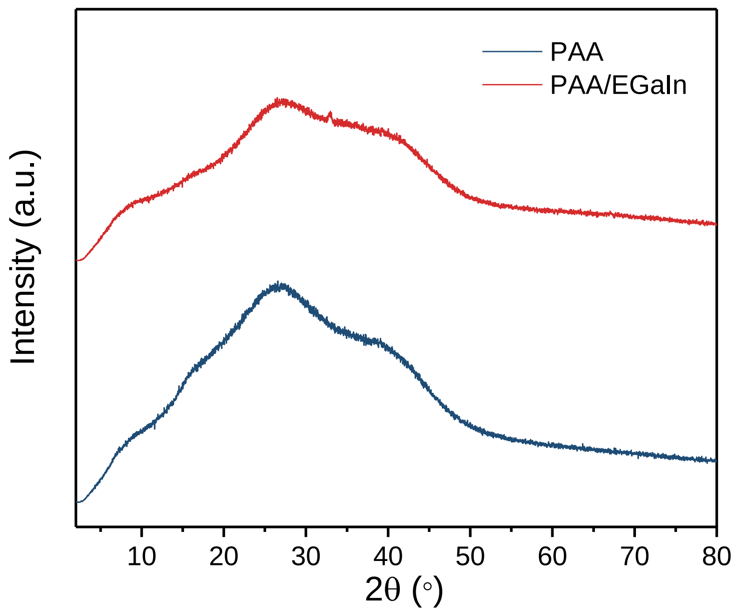

Supplementary Figure 6. XRD patterns of PAA foam and SMF.

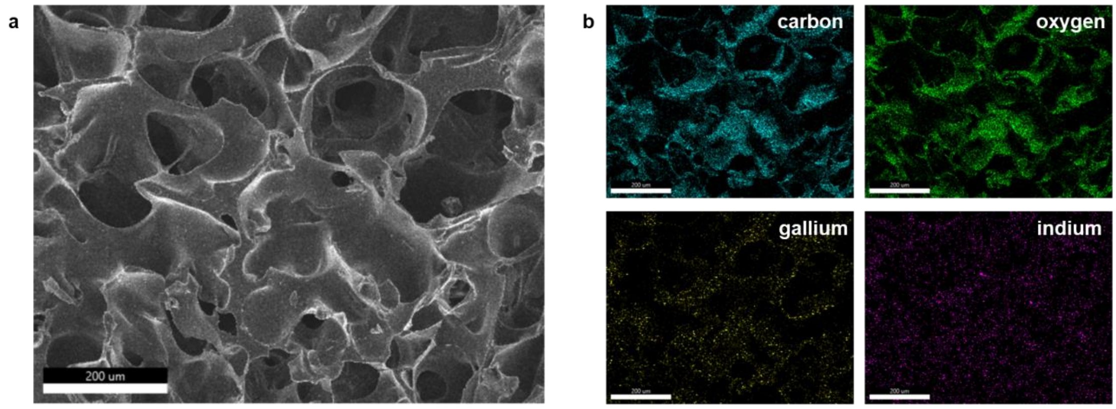

Supplementary Figure 7. a) Cross-section SEM image of SMF. b) EDS mapping of carbon element, oxygen element, gallium element and indium element in the cross-sectional SEM morphology, scale bar 200 μm.

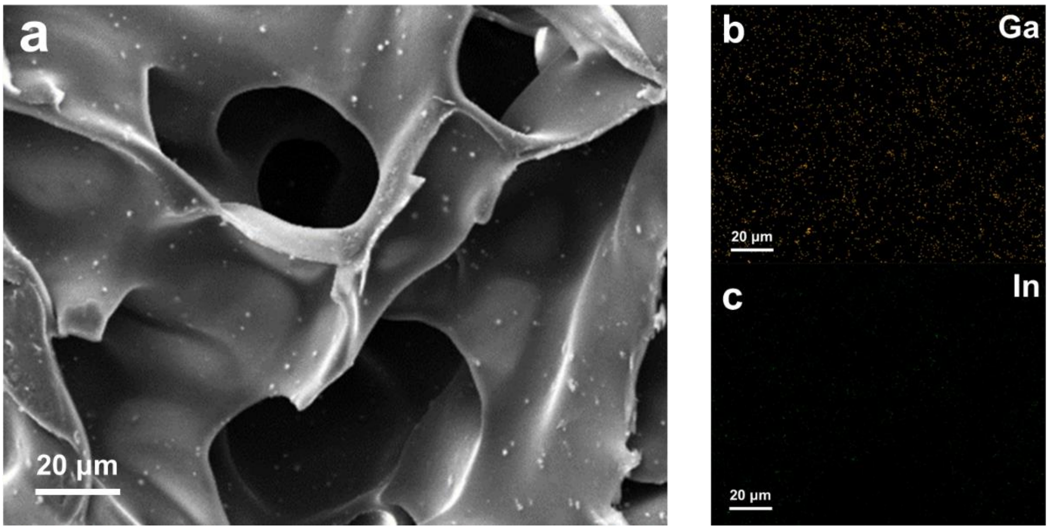

Supplementary Figure 8. a) Cross-section SEM image of SMF. EDS mapping of gallium element (b) and indium element (c) in the cross-sectional SEM morphology, scale bar 20 μm.

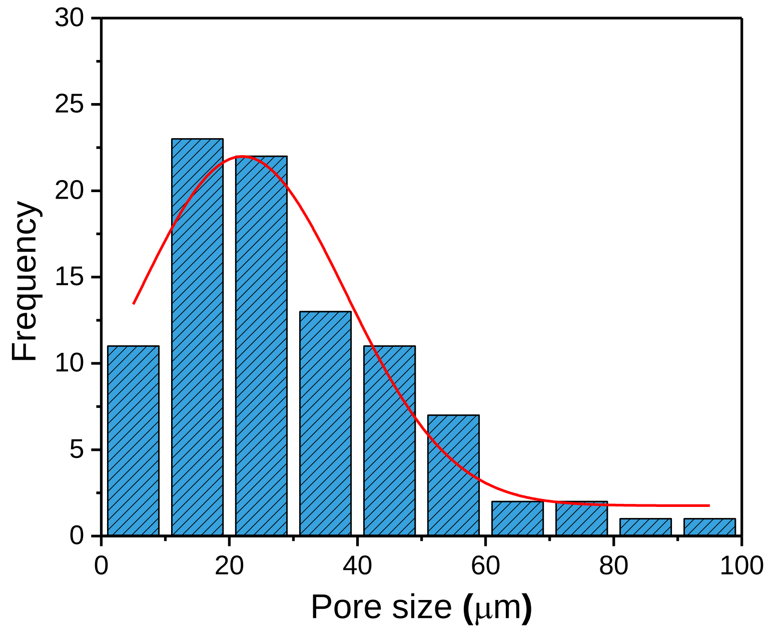

Supplementary Figure 9. Pore size distribution of SMF analyzed by SEM images.

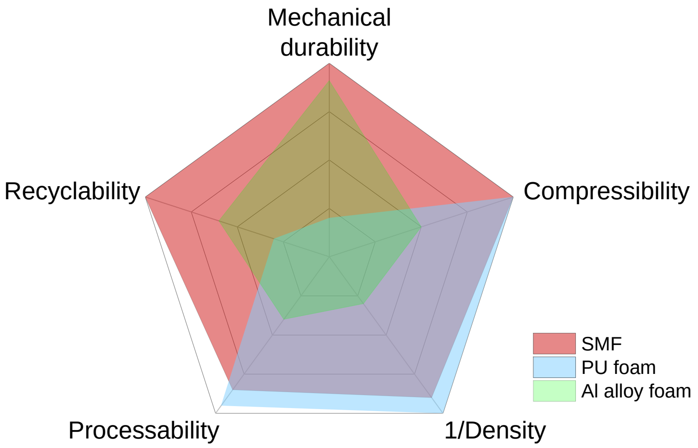

Supplementary Figure 10. Comparison of mehcanical durability, recyclability, processability, density and compressibility of SMF, PU foam and Al alloy foam based on Supplementary Table 1.

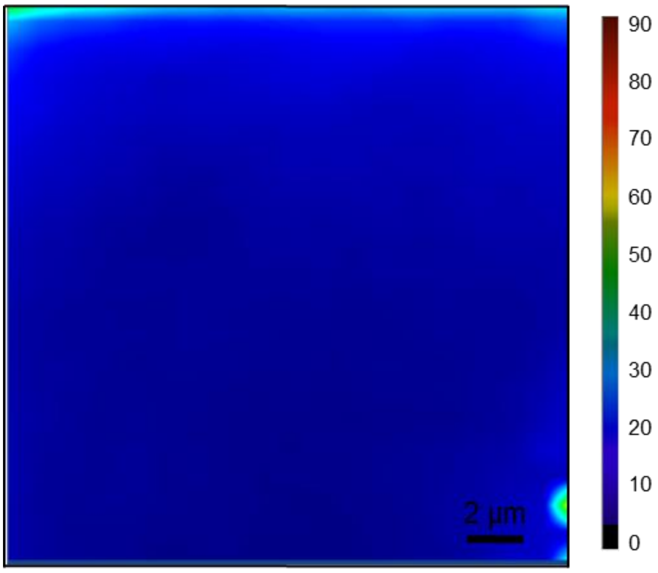

Supplementary Figure 11. 2D Raman intensity surface mapping at intensity of 1719 cm^{-1} of PAA foam

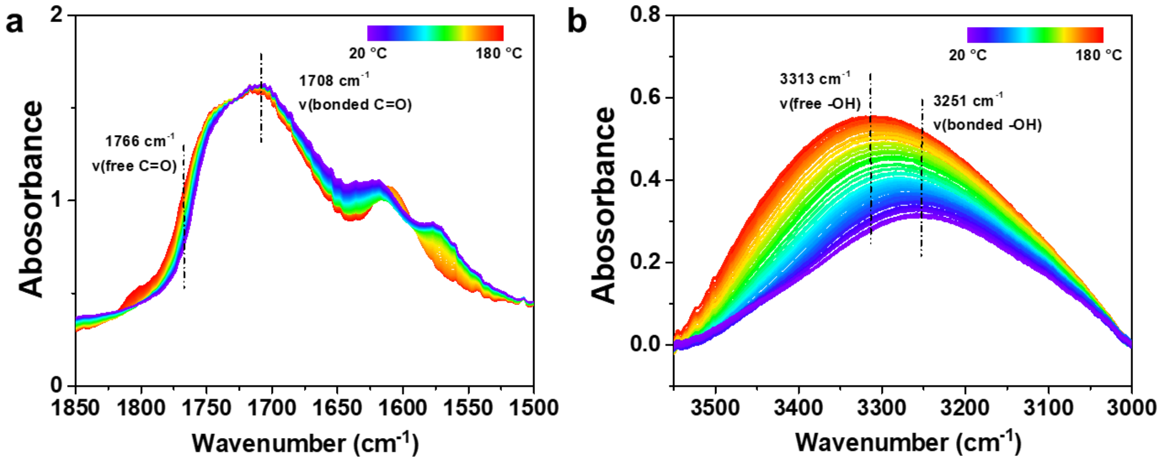

Supplementary Figure 12. Temperature-dependent FTIR spectra of SMF heating from 20 to 180 ˚C, 1850-1500 cm^{-1} (a), 3550-3000 cm^{-1} (b).

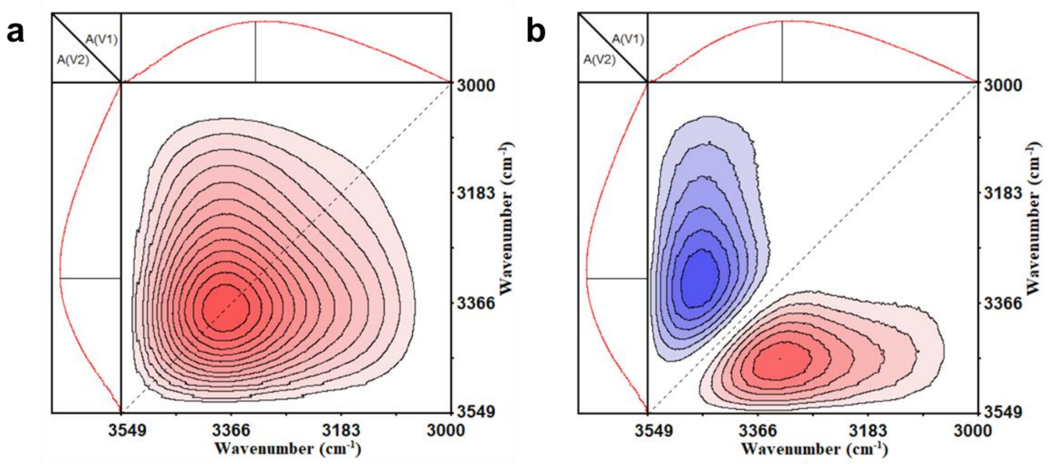

Supplementary Figure 13. Synchronous (a) and asynchronous (b) generalized 2D correlation spectra from 3550 to 3000 cm^{-1} of SMF during heating from 20 to 180 ℃.

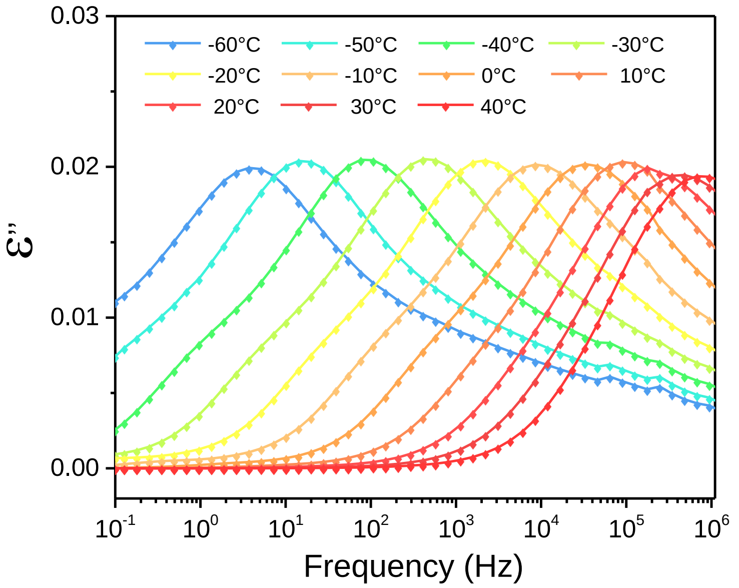

Supplementary Figure 14. Dielectric loss ε′′ as a function of frequency for PAA from -40 to 40 ˚C.

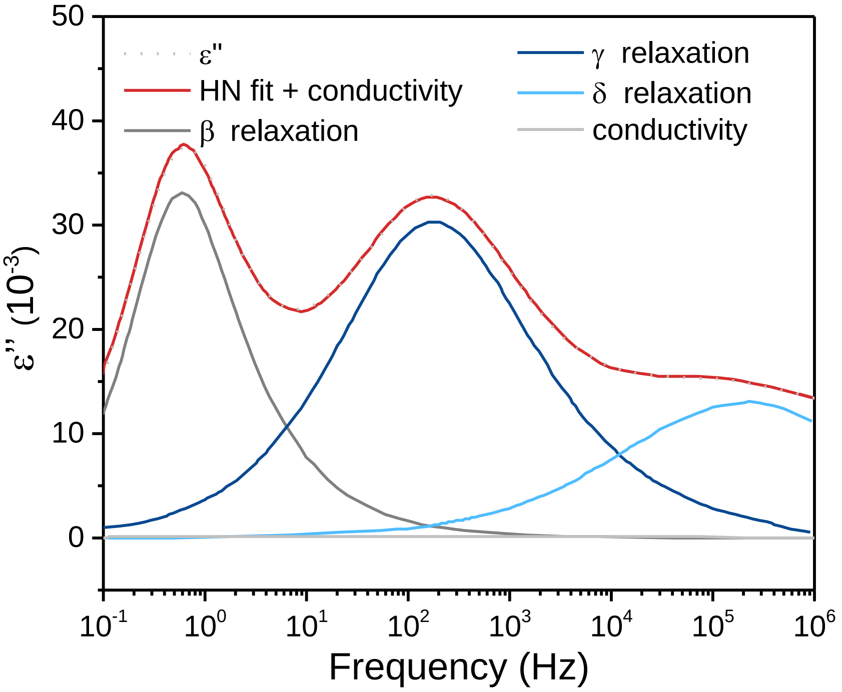

Supplementary Figure 15. Dielectric loss ε″ as a function of frequency fitted by a combination of three H-N equations at 0 °C.

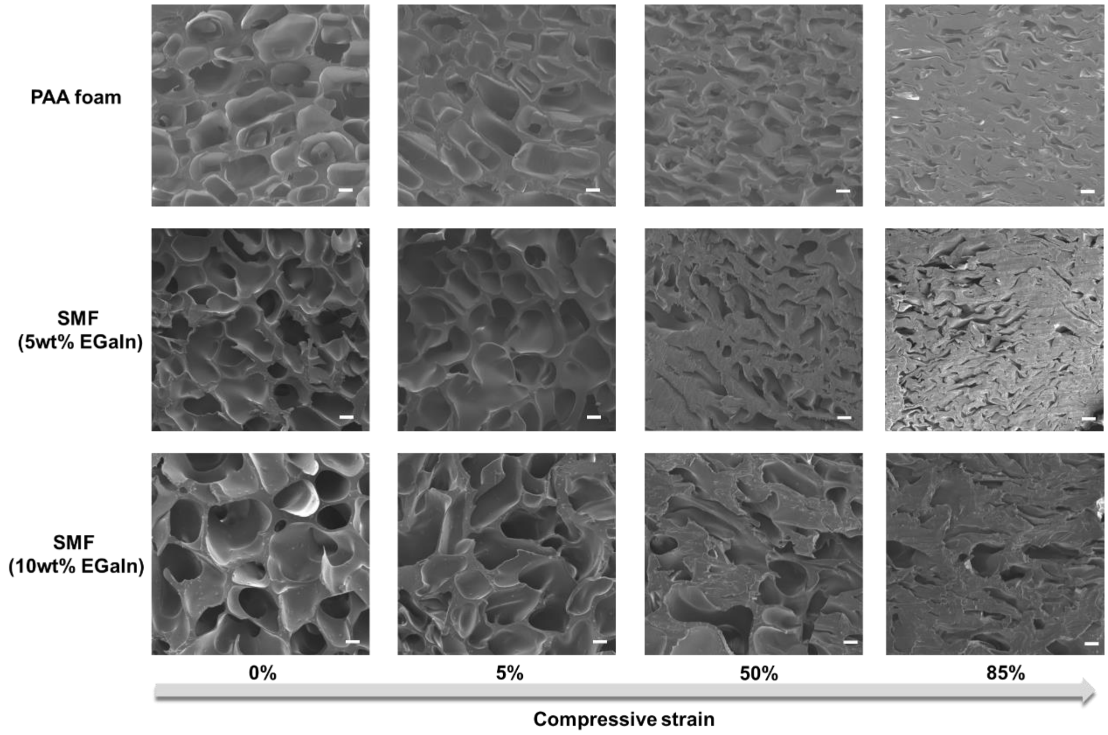

Supplementary Figure 16. Cross-section SEM image of PAA foam and SMFs (5 wt% and 10 wt% EGaIn) under compressive strain of 0%, 5%, 50% and 85%, Scale bar: 20 μm.

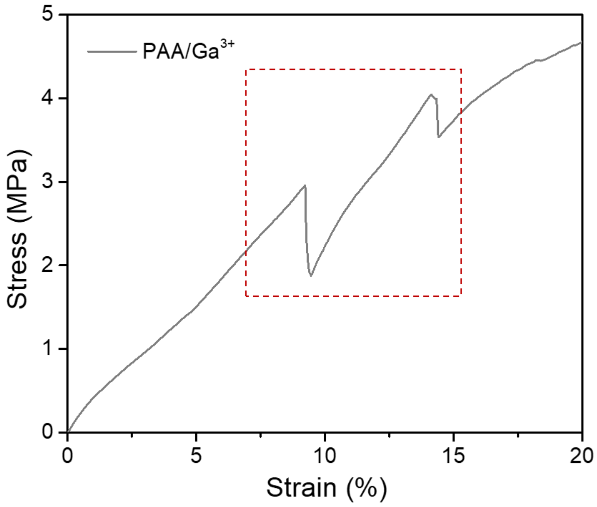

Supplementary Figure 17. Compressive stress-strain curve of Ga^{3+} crosslinked PAA foam.

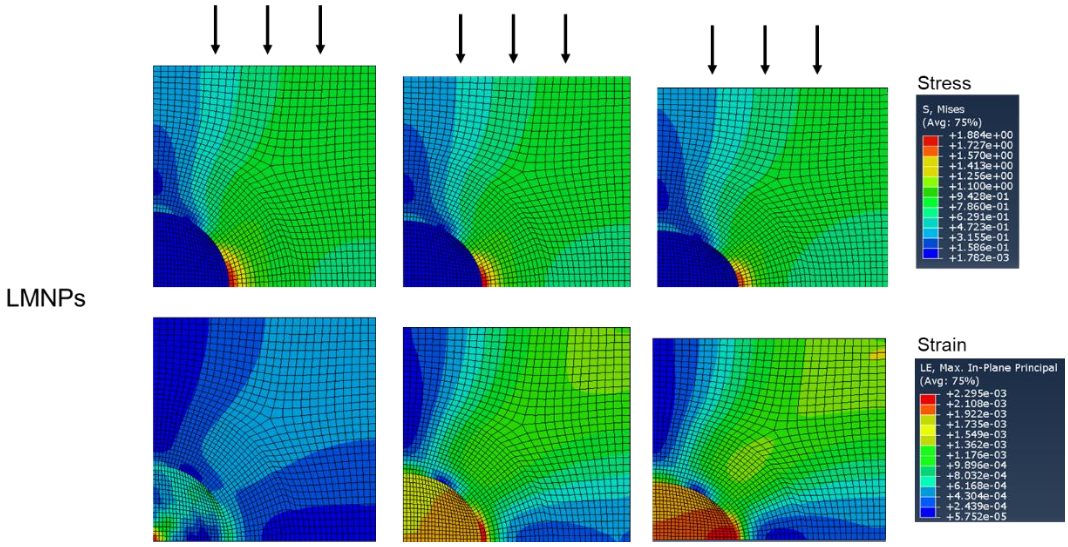

Supplementary Figure 18. Finite element analysis of the stress and strain distribution of PAA-LMNPs.

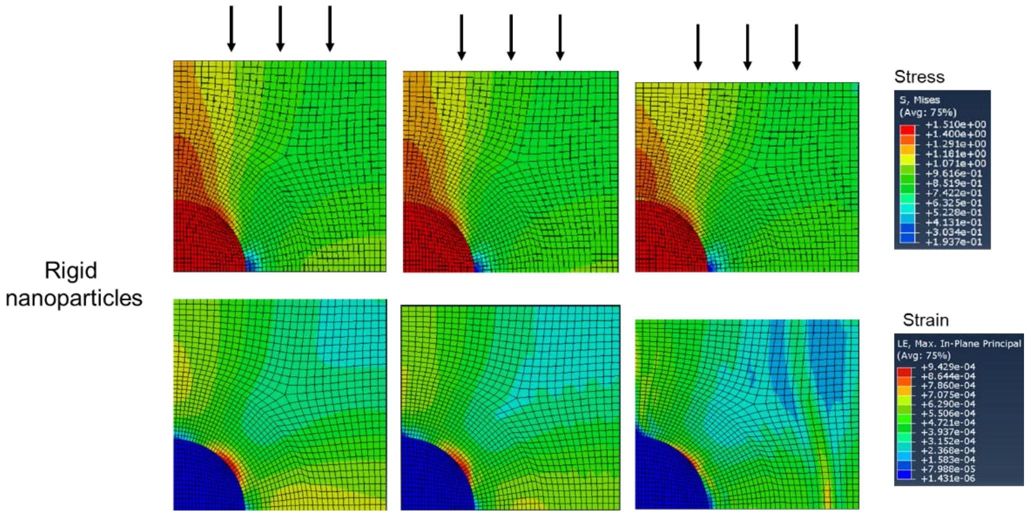

Supplementary Figure 19. Finite element analysis of the stress and strain distribution of PAA-Rigid nanoparticles.

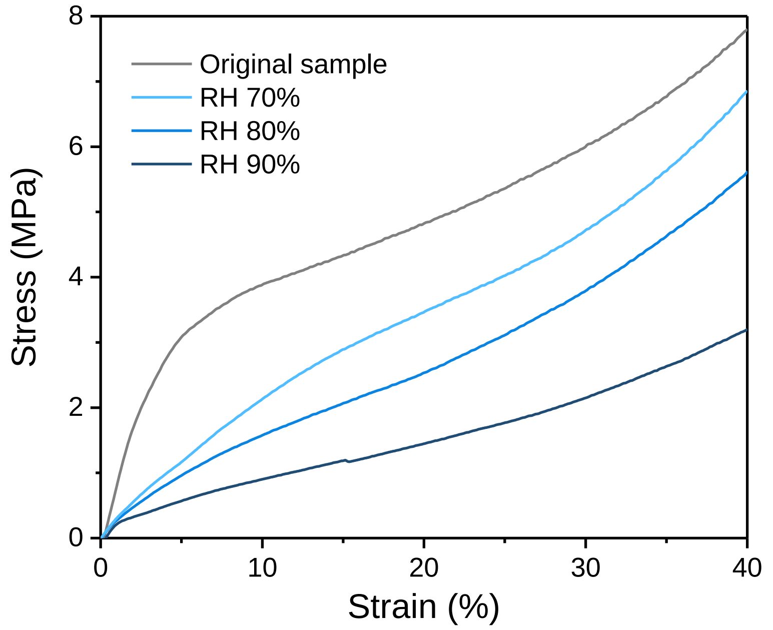

Supplementary Figure 20. Compressive stress-strain curve of SMFs with relative humidity of 70%, 80% and 90%.

Supplementary Table

Supplementary Table 1. Comparison of mechanical durability, recyclability, processability, density and compressibility of SMF, PU foam and Al alloy foam.

Type of foams	SMF	PU foam	Al alloy foam
Mechanical durability	Specific Modulus: 281.23 kN m kg^{-1}	Specific Modulus: ~46-68 kN m kg^{-1}	Specific Modulus: ~200 kN m kg^{-1}
Recyclability	Water reprocessing at 80 ˚C	Crosslinking structure. Hard to recycle.	Melting of scrap > 600 ˚C
Processability	Freeze-drying	Foaming and curing at 150 ˚C	Foaming at ~ 800 ˚C
Density	0.20-0.32 g cm^{-3}	~ 0.25 g cm^{-3}	0.43-0.8 g cm^{-3}
Compressibility	Compressive strain > 90%	Compressive strain > 90%	Brittle cracking at ~10% strain
Ref.	This work	[1,2]	[3,4]

Supplementary Table 2. Comparison of the specific strength and specific modulus of SMFs and other reported foams.

No.	Materials	Specific Modulus (kN m kg-1)	Specific Strength (kN m kg-1)	Ref.
1	SMF-1	39.56	145.55	This work
2	SMF-2	281.23	489.68	
3	SMF-3	214.36	342.66	
4	LigSi Aerogel	31.00	75.50	[5]
5		53.89	90.00	
6		376.32	75.79	
7		256.96	38.70	
8	DCPA-1	1.65	~8.71	[6]
9	DCPA-2	2.30	~25.96	
10	DCPA-3	2.45	~16.0	
11	Gelatin foam	24.53	~56.04	[7]
12	MIA-1	129.18	42.95	[8]
13	MIA-1-TEA	90.2	38.06	
14	Al99.5 0-100	33	~57.63	[9]
15	AlSi12 0-100	63	~70.59	
16	Alporas foam	~200	~35.92	[10]
17	Al foams	226	99.6	[11]
18	PUCc-10	150	7.2	[2]
19	PUCc-20	101.4	~5.4	
20	PUCa-10	65.0	~8.3	

21	PUCa-20	99.2	~8.7	
22	PS Foam	203	~11.6	[12]
23	polyHIPEs foams-1	22	2	
24	polyHIPEs foams-2	39	2	[13]
25	polyHIPEs foams-3	81	4	
30	SCD silica aerogel-1	~59.62	2.03	
31	SCD silica aerogel-2	~11.28	~6.60	[14]
32	SCD silica aerogel-3	~3.55	~27.60	
33	SiC NWA	~4.2	~3.2	[15]
34	SCD	~40.88	~70.72	[16]

Supplementary Note

Supplementary Note 1. Analyses of broadband dielectric measurements.

Below the glass transition temperature, the dielectric spectra were analyzed by Havriliak and Negami (H–N) function. The dielectric complex permittivity (ε*) as a function of frequency can be then described by the following formula:

$$\varepsilon^* = \varepsilon_\infty + \frac{\Delta\varepsilon}{[1 + (i\omega\tau\tau_{\mathrm{HN}})^\alpha]^\beta}$$

Where Δε is the dielectric strength, τ_{HN} is the characteristic relaxation time. The parameters α and β (0 < α, αβ ≤ 1) define the symmetrical and asymmetrical broadening of the loss peak.

The relationship between the average relaxation time (τ_{max}) and τ_{HN} can be expressed by the following equation:

$$\tau_{\max} = \tau_{\mathrm{HN}}\left[\sin\frac{\pi\alpha\beta}{2(1+\beta)}\right]^{-\frac{1}{\alpha}}\left[\sin\frac{\pi\alpha}{2(1+\beta)}\right]^{-\frac{1}{\alpha}};\ f_{\max} = \frac{1}{2\pi\tau_{\max}}$$

where f_{max} is the frequency at the maximum value of the dielectric loss ε''.

The relationship between temperature and τ_{max} can be described by the Arrhenius function:

$$\tau_{\max} = \tau_0 \exp\left(\frac{E_a}{RT}\right)$$

where E_a is the activation energy and τ_0 is a constant.

Supplementary references

1. Li, H., Sinha, T. K., Oh, J. S. & Kim, J. K. Soft and Flexible Bilayer Thermoplastic Polyurethane Foam for Development of Bioinspired Artificial Skin. *ACS Appl. Mater. Interfaces* **10**, 14008–14016 (2018).
2. Stanzione, M. *et al.* Tuning of polyurethane foam mechanical and thermal properties using ball-milled cellulose. *Carbohydr. Polym.* **231**, 115772 (2020).
3. Lin, Y., Zhang, Q., Ma, X. & Wu, G. Mechanical behavior of pure Al and Al–Mg syntactic foam composites containing glass cenospheres. *Compos. Part A Appl. Sci. Manuf.* **87**, 194–202 (2016).
4. Aldoshan, A. & Khanna, S. Effect of relative density on the dynamic compressive behavior of carbon nanotube reinforced aluminum foam. *Mater. Sci. Eng. A* **689**, 17–24 (2017).
5. Fan, Q. *et al.* Water-Induced Self-Assembly and In Situ Mineralization within Plant Phenolic Glycol-Gel toward Ultrastrong and Multifunctional Thermal Insulating Aerogels. *ACS Nano* **16**, 9062–9076 (2022).
6. Zhang, X. *et al.* Weldable and closed-loop recyclable monolithic dynamic covalent polymer aerogels. *Natl. Sci. Rev.* **9**, nwac012 (2022).
7. Cai, S. *et al.* Ultralong Organic Phosphorescent Foams with High Mechanical Strength. *J. Am. Chem. Soc.* **143**, 16256–16263 (2021).
8. Zhang, X. *et al.* Mechanically Interlocked Aerogels with Densely Rotaxanated Backbones. *J. Am. Chem. Soc.* **144**, 11434–11443 (2022).
9. Kemény, A., Leveles, B., Bubonyi, T. & Orbulov, I. N. Effect of particle size and volume ratio of ceramic hollow spheres on the mechanical properties of bimodal composite metal foams. *Compos. Part A Appl. Sci. Manuf.* **140**, 106152 (2021).
10. Rajaneesh, A., Sridhar, I. & Rajendran, S. Relative performance of metal and polymeric foam sandwich plates under low velocity impact. *Int. J. Impact Eng.* **65**, 126–136 (2014).
11. Jung, A., Pullen, A. D. & Proud, W. G. Strain-rate effects in Ni/Al composite metal foams from quasi-static to low-velocity impact behaviour. *Compos. Part A Appl. Sci. Manuf.* **85**, 1–11 (2016).
12. Aksit, M. *et al.* Extruded polystyrene foams with enhanced insulation and mechanical properties by a benzene-trisamide-based additive. *Polymers (Basel).* **11**, 268 (2019).
13. Haibach, K., Menner, A., Powell, R. & Bismarck, A. Tailoring mechanical properties of highly porous polymer foams: Silica particle reinforced polymer foams via emulsion templating. *Polymer* **47**, 4513–4519 (2006).
14. Iswar, S. *et al.* Dense and strong, but superinsulating silica aerogel. *Acta Mater.* **213**, 116959 (2021).
15. Su, L. *et al.* Ultralight, Recoverable, and High-Temperature-Resistant SiC Nanowire Aerogel. *ACS Nano* **12**, 3103–3111 (2018).
16. Guerrero-Alburquerque, N. *et al.* Strong, Machinable, and Insulating Chitosan-Urea Aerogels: Toward Ambient Pressure Drying of Biopolymer Aerogel Monoliths. *ACS Appl. Mater. Interfaces* **12**, 22037–22049 (2020).
